# Real-World Effectiveness of Tildrakizumab for Moderate-to-Severe Plaque Psoriasis in Canada

**DOI:** 10.1177/12034754241302827

**Published:** 2024-12-14

**Authors:** Mohannad Abu-Hilal, Jeff Cowger, Mohammed Bawazir, Dusan Sajic, Iryna Savinova, Belinda Yap, Rami El-Sayegh, Talshyn Bolatova, Pak Chan, Ajith Cy

**Affiliations:** 1 Division of Dermatology, McMaster University, Hamilton, ON, Canada; 2 Skin Therapy Centre, Advanced Medical Group, London, ON, Canada; 3 Falls Dermatology Centre, Niagara Falls, ON, Canada; 4 Alliance Clinical Trials and Probity Medical Research, Inc., Waterloo, ON, Canada; 5 Faculty of Health Sciences Department of Medicine, McMaster University, Waterloo, ON, Canada; 6 Guelph Dermatology Research, Guelph, ON, Canada; 7 Faculty of Health Sciences Michael G. DeGroote School of Medicine, McMaster University, Hamilton, ON, Canada; 8 Cencora, Innomar Strategies Inc., Oakville, ON, Canada; 9 Sun Pharma Canada Inc., Brampton, ON, Canada

**Keywords:** tildrakizumab, anti–IL-23, psoriasis, real-world

## Abstract

**Background:**

Tildrakizumab is an interleukin-23 inhibitor approved in Canada in 2021 for the treatment of adults with moderate-to-severe plaque psoriasis.

**Objectives:**

To evaluate real-world effectiveness of tildrakizumab for the treatment of moderate-to-severe plaque psoriasis in Canada.

**Methods:**

A multicenter, retrospective study was conducted in Canada in adults with moderate-to-severe plaque psoriasis for ≥1 year treated with tildrakizumab for ≥12 weeks. Effectiveness was evaluated from proportions of patients achieving ≥75%/≥90%/100% improvement from baseline in Psoriasis Area and Severity Index (PASI 75/90/100 response) and Physician Global Assessment (PGA) 0 or 1 at weeks 16 (±4), 24 (±8), and 48 (±12). Subgroup analyses were performed based on prior biologic use and special site involvement.

**Results:**

The study included 75 patients (mean age, 50.5 years; 52.0% female; 82.7% bio-naïve; 73.3% with special site involvement). Absolute mean (standard deviation) PASI score improved from 16.1 (6.7) at baseline to 1.3 (1.7) at the week 48 (91.7% improvement), 95.7%/69.6%/34.8% of patients achieved PASI 75/90/100 response, and 93.0% achieved PGA 0/1 at the week 48. In subgroup analyses, 94.7%/71.1%/34.2% of bio-naïve patients, 100.0%/62.5%/37.5% of bio-experienced patients, 100.0%/71.4%/28.6% of patients with special site involvement, and 81.8%/63.6%/54.6% of patients without special site involvement achieved PASI 75/90/100 response, and 87.5%, 94.3%, 97.0%, and 80.0% of patients, respectively, achieved PGA 0/1 at the week 48. None of the differences among subgroups were statistically significant; however, patient numbers were too small to support robust conclusions.

**Conclusions:**

Tildrakizumab is effective for the treatment of moderate-to-severe plaque psoriasis in adults in a real-world setting in Canada.

## Introduction

Psoriasis is a chronic, inflammatory skin disorder with an estimated global prevalence of 0.5% to 11.4% in adults, with plaque-type psoriasis being the most common form.^1-3^ While mild-to-moderate plaque psoriasis [3%-10% body surface area (BSA) affected] can be treated with topical agents, moderate-to-severe disease (>10% BSA affected) often requires treatment with systemic and/or biologic agents.^1,4^

Interleukin (IL)-23 is considered one of the master cytokines/regulators in the pathogenesis of psoriasis, making it a crucial therapeutic target.^5,6^ Tildrakizumab, a humanized monoclonal antibody that inhibits IL-23 by targeting the p19 subunit, was approved in Canada in 2021, as well as in several other countries, for the treatment of adults with moderate-to-severe plaque psoriasis who are candidates for systemic therapy or phototherapy.^7-9^ The efficacy and safety of tildrakizumab were evaluated in 2 Phase 3, randomized, double-blind clinical trials, reSURFACE 1 (NCT01722331) and reSURFACE 2 (NCT01729754). In both trials, significantly higher proportions of patients receiving tildrakizumab 100 mg vs placebo achieved ≥75%, ≥90%, and 100% improvements from baseline in the Psoriasis Area and Severity Index (PASI) score (PASI 75, PASI 90, and PASI 100 responses, respectively) and the Physician Global Assessment (PGA) response at the Week 12, and tildrakizumab treatment was well tolerated.^9^

Although randomized controlled trials provide valuable insights into the efficacy and safety of tildrakizumab, due to the stringent inclusion and exclusion criteria for patients’ participation, clinical trials may not fully represent the patient population or reflect the drug’s actual efficacy and safety in real-world patients. Although a number of previous studies provide evidence for the effectiveness and safety of tildrakizumab in real-world settings,^10-15^ the majority were conducted in Europe, and the results may not be generalizable to the real-world experiences of other regions and countries with their own distinct populations and practice patterns. Here, we address the relative lack of real-world data for tildrakizumab in North America and specifically in Canada by presenting the results of the first multicenter, retrospective, real-world study to evaluate the effectiveness of tildrakizumab for the treatment of moderate-to-severe plaque psoriasis in Canada.

## Patients and Methods

### Study Design

This was a multicenter, retrospective study. Adult patients with moderate-to-severe plaque psoriasis who were diagnosed for at least 1 year and had received tildrakizumab at the approved dose for at least 12 weeks were included in the study. The data were collected from January 2022 to June 30, 2023, across 5 dermatology centres in Ontario, Canada. Patients who did not complete treatment with tildrakizumab for at least 12 weeks were excluded. Patients concurrently undergoing treatment with other systemic therapies for psoriasis or psoriatic arthritis, including methotrexate, acitretin, or cyclosporine, were also excluded in order to provide an accurate assessment of the efficacy of tildrakizumab as a monotherapy, without the confounding influence of other systemic treatments.

Data extracted included demographics, duration of psoriasis, prior biologic experience, involvement of a special site (face, genitals, palms/soles, and scalp), and comorbidities, as well as the PASI score and the PGA score at baseline and when available around week 16, week 24, and week 48. The study was conducted in accordance with the principles of the Declaration of Helsinki. The study was approved by the Hamilton Integrated Research Ethics Board (number: 16387). All patients provided informed consent for the use of their records.

### Effectiveness Outcomes

Effectiveness outcomes included the mean change from baseline in PASI score and proportions of patients achieving PASI 75/90/100 responses and a PGA score of 0 (clear) or 1 (almost clear; PGA 0/1 response) at Weeks 16, 24, and 48.

### Statistical Analysis

Data were analyzed using a complete case approach. Data were extracted from patient visits within the specified windows around each time point. When multiple visits were available for the same patient within one of these windows [week 16 (±4 weeks), week 24 (±8 weeks), and week 48 (±12 weeks)], the visit closest to the reporting time point was used. Continuous variables were reported using frequency, mean, and standard deviation (SD). Categorical variables were reported as count and percentage. Absolute PASI scores, PASI 75/90/100 response rates, and PGA 0/1 response rates were assessed in the overall population and in subgroups of patients defined by prior biologic experience and special site involvement. Missing data were not imputed. Continuous variables were analyzed with Student’s t-test, and categorical variables were analyzed with chi-squared test (if possible) or Fisher’s exact test (if required by subgroup sizes).

## Results

### Baseline Demographics and Clinical Characteristics

Of 114 patients treated from January 2022 to June 30, 2023, 75 patients met eligibility criteria and were included in the study (Supplemental Figure 1). The patients’ mean ± SD age was 50.5 ± 18.1 years, 76.0% (57/75) were <65 years old, and 52.0% (39/75) were female (Supplemental Table 1). A majority of patients (55/75, 73.3%) had involvement of at least 1 special site (including face, genitals, palms/soles, and scalp), most frequently the scalp (50/75, 66.7%); 34.7% of patients had involvement of only 1 special site, and 38.7% had 2 or more special sites involved (Supplemental Table 1). The most common comorbidities were hypertension (19/75, 25.3%) and dyslipidemia (16/75, 21.3%; Supplemental Table 1).

Most of the patients (62/75, 82.7%) were naïve to biologic therapies (bio-naïve); the remaining patients were previously treated with biologics targeting tumour necrosis factor-alpha (TNF-α), IL-17, IL-23 p19, and IL-12/23 (bio-experienced; Supplemental Table 1). The absolute mean ± SD PASI score at baseline was 16.1 ± 6.7 and was comparable between bio-naïve and bio-experienced patients and between patients with or without involvement of special sites (Supplemental Table 1). Of the 75 patients, 74 (98.7%) had a PGA score of 3 (moderate) or 4 (severe) at baseline; 1 patient with a partial response to previous treatment with brodalumab had a baseline PGA score of 2 (mild; Supplemental Table 1).

### Effectiveness

**Figure 1.**
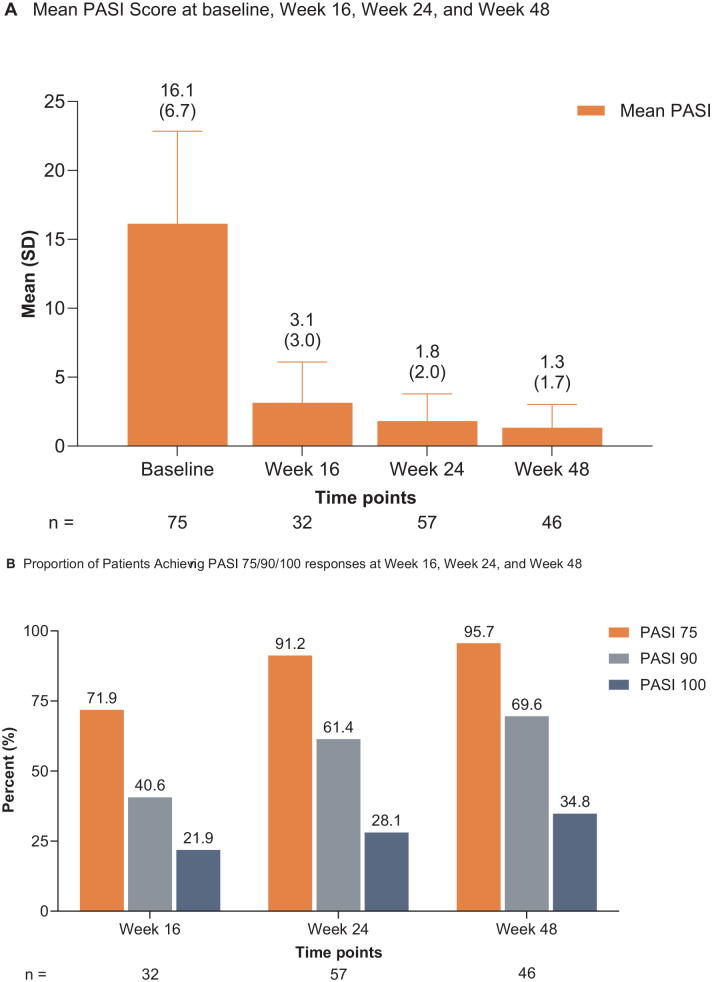
(A) Mean PASI scores and (B) proportions of patients achieving PASI 75/90/100 responses through week 48. PASI, Psoriasis Area and Severity Index; PASI 75/90/100 response, ≥75%/≥90%/100% improvement from baseline in PASI score; SD, standard deviation.

Absolute mean ± SD PASI score improved throughout the study from 16.1 ± 6.7 at baseline to 3.1 ± 3.0 at the week 16 (n = 32; 80.6% improvement), 1.8 ± 2.0 at the week 24 (n = 57; 88.8% improvement), and 1.3 ± 1.7 at the week 48 (n = 46; 91.7% improvement; Figure 1A). The proportions of patients achieving PASI 75/90/100 responses increased over time, with 95.7% achieving PASI 75 response, 69.6% achieving PASI 90 response, and 34.8% achieving PASI 100 response at the week 48 (Figure 1B). The proportion of patients with PGA 0/1 response also increased throughout the study to 93.0% (40/43) at the week 48 (Table 1).

**Table 1.** Proportions of Patients Achieving PGA 0/1 Through Week 48.

Time Point	Overall	Previous biologic therapy			Involvement of special site		
		Bio-naïve	Bio-experienced	*P*-value*	Yes	No	*P*-value*
Week 16 (n = 26)	18/26 (69.2)	14/19 (73.7)	4/7 (57.1)	.64	13/18 (72.2)	5/8 (62.5)	.67
Week 24 (n = 49)	43/49 (87.8)	38/42 (90.5)	5/7 (71.4)	.20	35/38 (92.1)	8/11 (72.7)	.12
Week 48 (n = 43)	40/43 (93.0)	33/35 (94.3)	7/8 (87.5)	.47	32/33 (97.0)	8/10 (80.0)	.13

Data are shown as n/N (%).

Abbreviation: PGA 0/1, Physician Global Assessment score of 0 (clear) or 1 (almost clear).

* Fisher’s exact test.

### Effectiveness of Tildrakizumab by Prior Biologic Therapy and Involvement of Special Sites

The mean ± SD PASI score improved from baseline to week 48 for both bio-naïve (16.3 ± 7.1 to 1.3 ± 1.8) and bio-experienced (15.3 ± 4.7 to 1.4 ± 1.3) patients, with no statistically significant difference between the subgroups (*P* = .98; Supplemental Table 2); there was also no statistically significant difference between bio-naïve and bio-experienced patients for the achievement of PASI 75 (94.7% vs 100.0%, *P* = 1.00), PASI 90 (71.1% vs 62.5%, *P* = .96), and PASI 100 (34.2% vs 37.5%, *P* = 1.00) responses at the week 48 (Supplemental Table 3). The mean ± SD PASI score improvement from baseline to week 48 was not significantly different between patients with special site involvement (15.8 ± 7.1 to 1.2 ± 1.2) and those with no special sites involved (17.0 ± 5.7 to 1.8 ± 2.8; *P* = .54; Supplemental Table 2). Similarly, there was no statistically significant difference between patients with vs without special site involvement for the achievement of PASI 75 (100.0% vs 81.8%; *P* = .08), PASI 90 (71.4% vs 63.6%; *P* = .91), and PASI 100 responses (28.6% vs 54.6%; *P* = .22) at the week 48 (Supplemental Table 4).

For the proportion of patients achieving PGA 0/1, there was no statistically significant difference between bio-naïve patients compared with bio-experienced patients (94.3% vs 87.5%, *P* = .47) or between patients with special site involvement relative to those without special site involvement (97.0% vs 80.0%, *P* = .13) at the week 48 (Table 1). No serious adverse events were observed in this study; adverse events recorded included mild sporadic cases of injection site reactions (2/75, 2.7%), nasopharyngitis (1/75, 1.3%), and upper respiratory tract infection (2/75, 2.7%).

## Discussion

Our study is the first to evaluate the real-world effectiveness of tildrakizumab in Canada. In this retrospective, real-world study, patients with moderate-to-severe plaque psoriasis treated with tildrakizumab achieved substantial improvement through week 48 based on the PASI and PGA scores. These results were consistent across subgroups defined by prior biologic use and special site involvement.

Our findings confirm the efficacy of tildrakizumab observed in the phase 3 reSURFACE 1 and reSURFACE 2 trials and in previous real-world studies with similar follow-up. The week 48 PASI 75/90/100 response rates of 95.7%/69.6%/34.8% in our study were comparable to the 91.2%/73.2%/34.4% (as observed) reported at the week 52 in the phase 3 trials.^16^ In other retrospective real-world studies, response rates at approximately 1 year ranged from 90% to 90.9% for PASI 75 response, 68.6% to 73.8% for PASI 90 response, and 58.7% to 62.7% for PASI 100 response.^10,11,14^ In a retrospective analysis of 42 patients with moderate-to-severe plaque psoriasis treated with tildrakizumab in Italy, mean ± SD PASI score decreased from 13.5 ± 5.9 at baseline to 2.9 ± 4.2 at week 52.^10^ In another Italian retrospective analysis of 237 patients with moderate-to-severe plaque psoriasis treated with tildrakizumab, an absolute PASI score ≤2 was observed in 86.9% of patients at week 52.^14^ In general, the rapid and sustained improvement in PASI scores during tildrakizumab treatment observed in our study was consistent with or better than results seen in the phase 3 trials and other real-world studies.

Our patients achieved clinical improvement regardless of prior biologic use and special site involvement. Although the number of patients with prior biologic experience was small, results were consistent with a pooled post hoc analysis of phase 2b and phase 3 trials of tildrakizumab in moderate-to-severe plaque psoriasis, in which the proportions of patients who achieved PASI 75 and PASI 90 responses were not significantly different between the bio-naïve and bio-experienced subgroups.^17^ Notably, although the phase 2 study excluded patients with prior exposure to 2 or more TNF-α inhibitors and the phase 3 studies excluded patients with prior exposure to IL-17 and IL-23 inhibitors (or the TNF-α inhibitor etanercept in reSURFACE 2 only),^9,18^ our study included patients who had previously used inhibitors of TNF-α, IL-17, IL-23 p19, and IL-12/IL-23. Therefore, our results suggest that patients with moderate-to-severe plaque psoriasis previously treated with other IL-23/T-helper 17 pathway inhibitors can still achieve clinical improvement when treated with tildrakizumab. We observed higher overall week 16 PASI 75/90 response rates in both bio-naïve and bio-experienced patients than the week 12 results from the phase 2b and phase 3 trials.^17^ Other 52 week real-world studies report either higher or lower PASI 75/90/100 response rates in bio-naïve vs bio-experienced patients, but the differences between groups were not generally statistically significant.^13,15^ Our results also align with the results from previous studies regarding the effectiveness and safety of tildrakizumab in patients with moderate-to-severe plaque psoriasis with special site involvement.^12,19,20^ Our study thus adds to the evidence that bio-experienced and bio-naïve patients as well as those with special site involvement can benefit from tildrakizumab treatment.

Results from our study suggest that the real-world effectiveness of tildrakizumab not only meets but exceeds expectations based on clinical trial data. Notably, our data provide additional information not readily available from the clinical trials. Published clinical trial subgroup analyses for bio-naïve vs bio-experienced patients report data only through the 12 week placebo-controlled period, at which time 63.6% and 56.0% of bio-naïve and bio-experienced patients, respectively, treated with tildrakizumab 100 mg achieved PASI 75 response.^17^ Furthermore, clinical trial results after week 28 were often reported only for PASI 75 responders because patients were re-randomized after week 28 based on their response levels; although 85% to 90% of patients treated with tildrakizumab 100 mg in reSURFACE 1 and reSURFACE 2 maintained PASI 75 response from week 28 through the end of 1 year of treatment (week 64/52), the proportion of all treated patients with response after a year is not available.^9^ The results of our subgroup analyses cannot be considered conclusive due to the small numbers of patients; however, our finding that 94.7% of bio-naïve patients achieved PASI 75 response by week 48 is new information and suggests that dermatologists and other prescribers should strongly consider tildrakizumab as a first-line biologic therapy. Similarly, clinical trial data for the efficacy of tildrakizumab for the treatment of psoriasis affecting special sites are available for the scalp but not for other difficult-to-treat areas.^21^ However, our study suggests that clinicians should still consider tildrakizumab for patients with involvement of these special sites, as the small number of patients with such involvement in our cohort still demonstrated favourable responses. Overall, our results support the principle that clinicians making treatment decisions for moderate-to-severe plaque psoriasis should consider not only clinical trial data but also real-world evidence from patients similar to those they see in practice.

Our study has some limitations. First, consistent with the retrospective nature of the study, data were not available for all patients at all time points. Second, the numbers of patients in the subgroup analyses by prior biologic therapy and special site involvement were small and do not support robust conclusions; therefore, we cannot be confident that the results are generalizable to all patients in these populations, particularly for bio-experienced patients and those with special site involvement. Future research including larger sample sizes, such as multicenter prospective studies or registry-based analyses, could enable more robust subgroup analyses and better detect any variability in treatment response based on factors such as prior biologic experience.

## Conclusion

Tildrakizumab is effective for the treatment of moderate-to-severe plaque psoriasis in adults treated in clinical practice in Canada, with increasing treatment response for up to 48 weeks. Only mild adverse events, such as injection site reactions, nasopharyngitis, and upper respiratory tract infections, were observed in our study, with no serious adverse events reported. These findings offer Canadian clinicians a valuable reference for considering tildrakizumab as a first-line biologic agent for patients with moderate-to-severe plaque psoriasis for whom IL-23 inhibitors are suitable, potentially influencing treatment choices. More real-world studies with larger populations may be needed to confirm the effectiveness and safety of tildrakizumab across the entire range of patient characteristics encountered in Canadian clinical practice.

## Supplemental Material

sj-docx-1-cms-10.1177_12034754241302827 – Supplemental material for Real-World Effectiveness of Tildrakizumab for Moderate-to-Severe Plaque Psoriasis in Canada Supplemental material, sj-docx-1-cms-10.1177_12034754241302827 for Real-World Effectiveness of Tildrakizumab for Moderate-to-Severe Plaque Psoriasis in Canada by Mohannad Abu-Hilal, Jeff Cowger, Mohammed Bawazir, Dusan Sajic, Iryna Savinova, Belinda Yap, Rami El-Sayegh, Talshyn Bolatova, Pak Chan and Ajith Cy in Journal of Cutaneous Medicine and Surgery

## References

[1] Menter A Strober BE Kaplan DH , et al. Joint AAD-NPF guidelines of care for the management and treatment of psoriasis with biologics. J Am Acad Dermatol. 2019;80(4):1029-1072. doi:10.1016/j.jaad.2018.11.057 30772098

[2] Michalek IM Loring B John SM . A systematic review of worldwide epidemiology of psoriasis. J Eur Acad Dermatol Venereol. 2017;31(2):205-212. doi:10.1111/jdv.13854 27573025

[3] Menter A Gottlieb A Feldman SR , et al. Guidelines of care for the management of psoriasis and psoriatic arthritis: Section 1. Overview of psoriasis and guidelines of care for the treatment of psoriasis with biologics. J Am Acad Dermatol. 2008;58(5):826-850. doi:10.1016/j.jaad.2008.02.039 18423260

[4] Kim WB Jerome D Yeung J . Diagnosis and management of psoriasis. Can Fam Physician. 2017;63(4):278-285. 28404701 PMC5389757

[5] Gooderham MJ Papp KA Lynde CW . Shifting the focus—the primary role of IL-23 in psoriasis and other inflammatory disorders. J Eur Acad Dermatol Venereol. 2018;32(7):1111-1119. doi:10.1111/jdv.14868 29438576 PMC6033004

[6] Yang K Oak ASW Elewski BE . Use of IL-23 inhibitors for the treatment of plaque psoriasis and psoriatic arthritis: a comprehensive review. Am J Clin Dermatol. 2021;22(2):173-192. doi:10.1007/s40257-020-00578-0 33301128 PMC7727454

[7] Ilumya™ (tildrakizumab) 100 mg/mL, for subcutaneous injection. Product Monograph. Sharjah, UAE: Sun Pharmaceutical Industries Limited; 2022.

[8] ILUMYA^®^ (tildrakizumab-asmn) injection, for subcutaneous use. Full Prescribing Information. Cranbury, NJ: Sun Pharmaceutical Industries, Inc.; 2024.

[9] Reich K Papp KA Blauvelt A , et al. Tildrakizumab versus placebo or etanercept for chronic plaque psoriasis (reSURFACE 1 and reSURFACE 2): results from two randomised controlled, phase 3 trials. Lancet. 2017;390(10091):276-288. doi:10.1016/S0140-6736(17)31279-5 28596043

[10] Ruggiero A Fabbrocicni G Cacciapuoti S Potestio L Gallo L Megna M . Tildrakizumab for the treatment of moderate-to-severe psoriasis: results from 52 weeks real-life retrospective study. Clin Cosmet Investig Dermatol. 2023;16:529-536. doi:10.2147/CCID.S402183 PMC9983574 36873660

[11] Di Brizzi EV Buononato D Benvenuto P , et al. Effectiveness and safety after a switch to tildrakizumab: a real world multicenter Italian study in psoriasis. Dermatol Pract Concept. 2023;13(4):e2023215. doi:10.5826/dpc.1304a215 PMC10656146 37992389

[12] Campione E Lambiase S Gaeta Shumak R , et al. A real-life study on the use of tildrakizumab in psoriatic patients. Pharmaceuticals (Basel). 2023;16(4):526. doi:10.3390/ph16040526 37111283 PMC10141711

[13] Wei NW Chi S Lebwohl MG . Retrospective analysis in patients with moderate to severe plaque psoriasis treated with tildrakizumab: real-life clinical data. J Psoriasis Psoriatic Arthritis. 2022;7(2):55-59. doi:10.1177/24755303221077211 39296827 PMC11361525

[14] Narcisi A Valenti M Gargiulo L , et al. Real-life effectiveness of tildrakizumab in chronic plaque psoriasis: a 52-week multicentre retrospective study-IL PSO (Italian landscape psoriasis). J Eur Acad Dermatol Venereol. 2023;37(1):93-103. doi:10.1111/jdv.18594 36156312 PMC10092064

[15] Graier T Weger W Jonak C , et al. Real-world effectiveness of anti-interleukin-23 antibodies in chronic plaque-type psoriasis of patients from the Austrian Psoriasis Registry (PsoRA). Sci Rep. 2022;12(1):15078. doi:10.1038/s41598-022-18790-9 36064563 PMC9442573

[16] Reich K Warren RB Iversen L , et al. Long-term efficacy and safety of tildrakizumab for moderate-to-severe psoriasis: pooled analyses of two randomized phase III clinical trials (reSURFACE 1 and reSURFACE 2) through 148 weeks. Br J Dermatol. 2020;182(3):605-617. doi:10.1111/bjd.18232 31218661 PMC7064936

[17] Poulin Y Ramon M Rosoph L , et al. Efficacy of tildrakizumab by patient demographic and disease characteristics across a phase 2b and 2 phase 3 trials in patients with moderate-to-severe chronic plaque psoriasis. J Eur Acad Dermatol Venereol. 2020;34(7):1500-1509. doi:10.1111/jdv.16187 31919889

[18] Papp K Thaçi D Reich K , et al. Tildrakizumab (MK-3222), an anti-interleukin-23p19 monoclonal antibody, improves psoriasis in a phase IIb randomized placebo-controlled trial. Br J Dermatol. 2015;173(4):930-939. doi:10.1111/bjd.13932 26042589

[19] Galluzzo M Talamonti M Cioni A , et al. Efficacy of tildrakizumab for the treatment of difficult-to-treat areas: scalp, nail, palmoplantar and genital psoriasis. J Clin Med. 2022;11(9):2631. doi:10.3390/jcm11092631 35566756 PMC9100809

[20] Megna M Tommasino N Potestio L , et al. Real-world practice indirect comparison between guselkumab, risankizumab, and tildrakizumab: results from an Italian 28-week retrospective study. J Dermatolog Treat. 2022;33(6):2813-2820. doi:10.1080/09546634.2022.2081655 35603992

[21] Gebauer K Spelman L Yamauchi PS , et al. Efficacy and safety of tildrakizumab for the treatment of moderate-to-severe plaque psoriasis of the scalp: a multicenter, randomized, double-blind, placebo-controlled, Phase 3b study. J Am Acad Dermatol. 2024;91(1):91-99. doi:10.1016/j.jaad.2024.03.025 38554938

