# Supplementary material for: Real-World Effectiveness of Tildrakizumab for Moderate-to-Severe Plaque Psoriasis in Canada
Source: J Cutan Med Surg. 2024 Dec 14;29(2):137–42. doi: 10.1177/12034754241302827 (PMC11979299; doi:10.1177/12034754241302827)
Supplement: sj-docx-1-cms-10.1177_12034754241302827 – Supplemental material for Real-World Effectiveness of Tildrakizumab for Moderate-to-Severe Plaque Psoriasis in Canada [file sj-docx-1-cms-10.1177_12034754241302827.docx]

**Supplemental Materials**

Table of Contents

[**Supplemental Table 1.** Baseline Demographics and Clinical Characteristics 2](#_Toc181177974)

[**Supplemental Table 2.** Subgroup Analysis of Disease Activity Improvement Based on PASI Score Through Week 48 5](#_Toc181177975)

[**Supplemental Table 3**. Analysis of Disease Activity Improvement by Prior Biologic Therapy Status Based on PASI Score Through Week 48 6](#_Toc181177976)

[**Supplemental Table 4.** Analysis of Disease Activity Improvement by Special Site Involvement Based on PASI Score Through Week 48 7](#_Toc181177977)

[**Supplemental Figure 1.** Patient Flow Diagram 8](#_Toc181177978)

## **Supplemental Table 1.** Baseline Demographics and Clinical Characteristics

| Characteristic | Tildrakizumab (n = 75) |
| --- | --- |
| Sex |  |
| Male | 36 (48.0) |
| Female | 39 (52.0) |
| Age, years, mean ± SD | 50.5 ± 18.1 |
| Age category |  |
| ≤65 years | 57 (76.0) |
| 20–35 years | 19 (25.3) |
| 36–45 years | 12 (16.0) |
| 46–55 years | 12 (16.0) |
| 56–65 years | 14 (18.7) |
| >65 years | 18 (24.0) |
| Duration of psoriasis |  |
| ≤5 years | 26 (34.7) |
| >5 to ≤10 years | 18 (24.0) |
| >10 to ≤15 years | 12 (16.0) |
| >15 to ≤20 years | 13 (17.3) |
| >20 years | 6 (8.0) |
| Involvement of special site |  |
| Yes | 55 (73.3) |
| 1 site affected | 26 (34.7) |
| >1 site affected | 29 (38.7) |
| No | 20 (26.7) |
| Type of special site involved |  |
| Face | 24 (32.0) |
| Genitals | 10 (13.3) |
| Palms and soles | 12 (16.0) |
| Scalp | 50 (66.7) |
| Previous biologic experience |  |
| Bio-naïve | 62 (82.7) |
| Bio-experienced | 13 (17.3) |
| Previous biologic treatments, n^a^ |  |
| Adalimumab | 4 |
| Brodalumab | 4 |
| Etanercept | 1 |
| Guselkumab | 3 |
| Ixekizumab | 2 |
| Risankizumab | 3 |
| Secukinumab | 1 |
| Ustekinumab | 2 |
| PASI, mean ± SD | 16.1 ± 6.7 |
| Bio-naïve | 16.3 ± 7.1 |
| Bio-experienced | 15.3 ± 4.7 |
| Special site involved | 15.8 ± 7.1 |
| No special site involved | 17.0 ± 5.7 |
| PGA 0/1 | 0 |
| PGA score of 3 or 4 | 74 (98.7) |
| Comorbidities |  |
| Previous malignancy^b^ | 2 (2.7) |
| Diabetes mellitus | 11 (14.7) |
| Depression | 10 (13.3) |
| Dyslipidemia | 16 (21.3) |
| Hypertension | 19 (25.3) |
| Infections^c^ | 4 (5.3) |
| Latent tuberculosis | 2 (2.7) |
| Psoriatic arthritis | 10 (13.3) |

Data are shown as n (%) unless otherwise specified.

^a^Some patients received more than 1 prior biologic treatment. ^b^Included colon cancer (n = 1) diagnosed in 2014 and in remission since 2016 and laryngeal cancer (n = 1) diagnosed in 2007 and in remission since 2009s. ^c^Included nasopharyngitis and upper respiratory tract infections.

PASI, Psoriasis Area and Severity Index; PGA, Physician Global Assessment; PGA 0/1, PGA score of 0 (clear) or 1 (almost clear); PGA >2, PGA score of >2 (more than “mild” in severity); SD, standard deviation.

## **Supplemental Table 2.** Subgroup Analysis of Disease Activity Improvement Based on PASI Score Through Week 48

|  | **Absolute PASI, mean ± SD** | | | | | |
| --- | --- | --- | --- | --- | --- | --- |
|  | **Previous biologic therapy** | | | **Involvement of special site** | | |
|  | **Bio-naïve** | **Bio-experienced** | ***P*-value*** | **Yes** | **No** | ***P*-value*** |
| Week 16 | 3.3 ± 3.1 (n = 24) | 2.8 ± 2.7 (n = 8) | 0.61 | 3.3 ± 2.6 (n = 22) | 2.7 ± 3.7 (n = 10) | 0.56 |
| Week 24 | 1.9 ± 2.0 (n = 49) | 1.4 ± 1.6 (n = 8) | 0.53 | 1.6 ± 1.6 (n = 44) | 2.6 ± 3.0 (n = 13) | 0.46 |
| Week 48 | 1.3 ± 1.8 (n = 38) | 1.4 ± 1.3 (n = 8) | 0.98 | 1.2 ± 1.2 (n = 35) | 1.8 ± 2.8 (n = 11) | 0.54 |

Data are shown as mean ± SD.

*Student’s t-test.

PASI, Psoriasis Area and Severity Index; SD, standard deviation.

### **Supplemental Table 3**. Analysis of Disease Activity Improvement by Prior Biologic Therapy Status Based on PASI Score Through Week 48

| **Relative PASI** | **Week 16** | | | **Week 24** | | | **Week 48** | | |
| --- | --- | --- | --- | --- | --- | --- | --- | --- | --- |
|  | **Bio-naïve** | **Bio-experienced** | ***P*-value*** | **Bio-naïve** | **Bio-experienced** | ***P*-value*** | **Bio-naïve** | **Bio-**  **experienced** | ***P*-value*** |
| PASI 75 | 17/24 (70.8) | 6/8 (75.0) | 1.00 | 44/49 (89.8) | 8/8 (100) | 0.79 | 36/38 (94.7) | 8/8 (100) | 1.00 |
| PASI 90 | 9/24 (37.5) | 4/8 (50.0) | 0.84 | 30/49 (61.2) | 5/8 (62.5) | 1.00 | 27/38 (71.1) | 5/8 (62.5) | 0.96 |
| PASI 100 | 6/24 (25.0) | 1/8 (12.5) | 0.81 | 13/49 (26.5) | 3/8 (37.5) | 0.83 | 13/38 (34.2) | 3/8 (37.5) | 1.00 |

Data are shown as n/N (%).

*Chi square test.

PASI 75/90/100, ≥75%/≥90%/100% improvement from baseline in Psoriasis Area and Severity Index; SD, standard deviation.

## **Supplemental Table 4.** Analysis of Disease Activity Improvement by Special Site Involvement Based on PASI Score Through Week 48

| **Relative PASI** | **Week 16** | | | **Week 24** | | | **Week 48** | | |
| --- | --- | --- | --- | --- | --- | --- | --- | --- | --- |
|  | **Special site** | **No special site** | ***P*-value*** | **Special site** | **No special site** | ***P*-value*** | **Special site** | **No special site** | ***P*-value*** |
| PASI 75 | 15/22 (68.2) | 8/10 (80.0) | 0.79 | 41/44 (93.2) | 11/13 (84.6) | 0.69 | 35/35 (100) | 9/11 (81.8) | 0.08 |
| PASI 90 | 7/22 (31.8) | 6/10 (60.0) | 0.26 | 28/44 (63.6) | 7/13 (53.8) | 0.75 | 25/35 (71.4) | 7/11 (63.6) | 0.91 |
| PASI 100 | 4/22 (18.2) | 3/10 (30.0) | 0.77 | 13/44 (29.3) | 3/13 (23.1) | 0.92 | 10/35 (28.6) | 6/11 (54.6) | 0.22 |

Data are shown as n/N (%).

*Chi square test.

PASI 75/90/100, ≥75%/≥90%/100% improvement from baseline in Psoriasis Area and Severity Index; SD, standard deviation.

## **Supplemental Figure 1.** Patient Flow Diagram

Patients assessed for eligibility (n = 114)

94 patients with ongoing tildrakizumab treatment status

20 patients excluded

20 did not initiate treatment with tildrakizumab

2 patients lost to follow-up

92 patients with follow-up

76 patients treated with tildrakizumab for at least 12 weeks

16 patients excluded

13 treated with tildrakizumab for <12 weeks

3 with unknown treatment duration

75 patients with absolute PASI ≥5 at baseline or special site involvement

1 patient excluded

1 with absolute PASI <5 and no special site involvement

PASI, Psoriasis Area and Severity Index.
